
JOURNAL INFORMATION
==============================
NLM Title Abbreviation: ScientificWorldJournal
Journal ID: ScientificWorldJournal
NLM Journal ID: 101131163
Journal ID: TSWJ

The Scientific World Journal

ISSN: 2356-6140
EISSN: 1537-744X
Publisher: Wiley

ARTICLE INFORMATION
==============================
PMCID: PMC6084418
DOI: 10.1100/tsw.2001.414
Article ID: 704824
Article version: 1
Subjects: Extended Abstract

Cgrp Release and Synergistic Interactions with Nitric Oxide: Implications for Pathogenesis of Septic Shock and the Vascular Problems of Diabetes Mellitus and Aging

Fiscus R.R. 1 2
Wang X. 1 2
Chan G.H.H. 1 2
Ma A.C.Y. 1 2
Chan S.L. 1 2
1 Department of Physiology, Faculty of Medicine, Epithelial Cell Biology Research Center, and The Center for Gerontology & Geriatrics, The Chinese University of Hong Kong, Shatin, NT, Hong Kong
2 Department of Physiology, The Peking University, Beijing, China
Publication date: 2001
Electronic publication date: 2001 Nov 7
Volume: 1
First page: 2
Last page: 2
Copyright: Copyright © 2001 R.R. Fiscus et al.
Copyright year: 2001
License: This is an open access article distributed under the Creative Commons Attribution License, which permits unrestricted use, distribution, and reproduction in any medium, provided the original work is properly cited.
License URL: https://creativecommons.org/licenses/by/3.0/

==============================
4th International Meeting on Calcitonin Gene-Related Peptide (CGRP)
*TheScientificWorld* (2001) 1(S1), 2
ISSN 1532-2246; DOI 10.1100/tsw.2001.414

### CGRP RELEASE AND SYNERGISTIC INTERACTIONS WITH NITRIC OXIDE: IMPLICATIONS FOR PATHOGENESIS OF SEPTIC SHOCK AND THE VASCULAR PROBLEMS OF DIABETES MELLITUS AND AGING

R.R. Fiscus, X. Wang, G.H.H. Chan, A.C.Y. Ma, and S.L. Chan
Department of Physiology, Faculty of Medicine, Epithelial Cell Biology Research Center, and The Center for Gerontology & Geriatrics, The Chinese University of Hong Kong, Shatin, NT, Hong Kong, and Department of Physiology, The Peking University, Beijing, China

Calcitonin gene-related peptide (CGRP) plays an important role as a vasodilator in local inflammation and response to injury. Various inflammatory mediators, including bradykinin (BK), histamine, and prostaglandins (PGs), trigger CGRP release from perivascular nerves, causing vasodilation. Elevated CGRP release is also thought to contribute to pathology of septic shock in human patients and endotoxemia (ETX) in animal models. ETX-induced CGRP release is partially blocked by cyclo-oxygenase inhibitors, suggestion role of PGs. Furthermore, other septic-shock mediators, such as BK and lactic acid, act synergistically to cause high levels of CGRP release[1].

In rat aortic rings, CGRP causes endothelium-dependent vasorelaxations and elevations of both cAMP and cGMP, all blocked by nitric oxide (NO) inhibitors[2], indicating NO involvement. Exogenous NO synergistically enhances vasorelaxant and cAMP responses to CGRP in endothelium-denuded aortic rings[3], suggesting endothelium-dependence of CGRPs responses involves NO potentiating direct (smooth muscle) effects of CGRP. These finding are especially relevant to septic shock, which involves elevated release of both CGRP and NO.

Recent studies from our laboratory show that both vasorelaxant responses *in vitro* and hypotensive responses *in vivo* to CGRP are severely impaired in elderly rats and young diabetic rats. These impairments may involve diminished NO release, known to occur in aging and diabetes, and damaging effects of glucose. Because endogenous CGRP is thought to serve as both antiatherogenic and antihypertensive agents, loss of these protective effects of CGRP during aging or diabetes may increase risks of developing atherosclerosis and hypertension.

### REFERENCES

1. Wang, X. and Fiscus, R.R. (1997) Am. J. Physiol. 273, E92–E98.
2. Fiscus, R.R. et al. (1991) Neuropeptides 20, 133–143.
3. Fiscus, R.R. et al. (1994) Neuropeptides 26, 133–144.
